# Functional bio-inspired hybrid fliers with separated ring and leading edge vortices

**DOI:** 10.1093/pnasnexus/pgae110

**Published:** 2024-03-11

**Authors:** Jin-Tae Kim, Hong-Joon Yoon, Shyuan Cheng, Fei Liu, Soohyeon Kang, Shashwot Paudel, Donghwi Cho, Haiwen Luan, Minkyu Lee, Gooyoon Jeong, Jaehong Park, Yu-Ting Huang, Su Eon Lee, Min Cho, Geonhee Lee, Mengdi Han, Bong Hoon Kim, Jinhui Yan, Yoonseok Park, Sunghwan Jung, Leonardo P Chamorro, John A Rogers

**Affiliations:** Department of Mechanical Engineering, Pohang University of Science and Technology, Pohang 37673, Republic of Korea; Department of Electronic Engineering, Gachon University, Gyeonggi-do 13120, Republic of Korea; Department of Mechanical Science and Engineering, University of Illinois, Urbana, IL 61801, USA; Querrey Simpson Institute for Bioelectronics, Northwestern University, Evanston, IL 60208, USA; Department of Mechanical Science and Engineering, University of Illinois, Urbana, IL 61801, USA; Department of Civil and Environmental Engineering, University of Illinois, Urbana, IL 61801, USA; Advanced Materials Division, Korea Research Institute of Chemical Technology, Daejeon 34114, Republic of Korea; Querrey Simpson Institute for Bioelectronics, Northwestern University, Evanston, IL 60208, USA; Querrey Simpson Institute for Bioelectronics, Northwestern University, Evanston, IL 60208, USA; Department of Advanced Materials Engineering for Information and Electronics, Integrated Education Institute for Frontier Science & Technology (BK21 Four), Kyung Hee University, Yongin-si, 17104, Republic of Korea; Department of Chemical and Biomolecular Engineering, University of Illinois, Urbana, IL 61801, USA; Querrey Simpson Institute for Bioelectronics, Northwestern University, Evanston, IL 60208, USA; Department of Robotics and Mechatronics Engineering, Daegu Gyeongbuk Institute of Science and Technology (DGIST), Daegu 42988, Republic of Korea; Department of Chemical and Biomolecular Engineering, University of Illinois, Urbana, IL 61801, USA; Advanced Materials Division, Korea Research Institute of Chemical Technology, Daejeon 34114, Republic of Korea; Department of Biomedical Engineering, College of Future Technology, Peking University, Beijing 100091, China; Department of Robotics and Mechatronics Engineering, Daegu Gyeongbuk Institute of Science and Technology (DGIST), Daegu 42988, Republic of Korea; Department of Civil and Environmental Engineering, University of Illinois, Urbana, IL 61801, USA; Department of Advanced Materials Engineering for Information and Electronics, Integrated Education Institute for Frontier Science & Technology (BK21 Four), Kyung Hee University, Yongin-si, 17104, Republic of Korea; Department of Biological and Environmental Engineering, Cornell University, Ithaca, NY 14853, USA; Department of Mechanical Science and Engineering, University of Illinois, Urbana, IL 61801, USA; Querrey Simpson Institute for Bioelectronics, Northwestern University, Evanston, IL 60208, USA

**Keywords:** bio-inspired design, aerodynamics, fluid mechanics, 3D fabrication, soft electronics

## Abstract

Recent advances in passive flying systems inspired by wind-dispersed seeds contribute to increasing interest in their use for remote sensing applications across large spatial domains in the Lagrangian frame of reference. These concepts create possibilities for developing and studying structures with performance characteristics and operating mechanisms that lie beyond those found in nature. Here, we demonstrate a hybrid flier system, fabricated through a process of controlled buckling, to yield unusual geometries optimized for flight. Specifically, these constructs simultaneously exploit distinct fluid phenomena, including separated vortex rings from features that resemble those of dandelion seeds and the leading-edge vortices derived from behaviors of maple seeds. Advanced experimental measurements and computational simulations of the aerodynamics and induced flow physics of these hybrid fliers establish a concise, scalable analytical framework for understanding their flight mechanisms. Demonstrations with functional payloads in various forms, including bioresorbable, colorimetric, gas-sensing, and light-emitting platforms, illustrate examples with diverse capabilities in sensing and tracking.

**Significance Statement**

Recent advances in the engineering of passive flying systems, inspired by wind-dispersed seeds, are of growing interest in the context of environmental and atmospheric monitoring. In this study, we showcase a class of hybrid passive flier created through a sequential controlled buckling process. These unusual structures, optimized by experimental, computational, and theoretical approaches, exploit intricate fluid phenomena, including the combination of separated vortex rings inspired by dandelion seeds and the leading-edge vortices (LEVs) observed in maple seeds. Representative examples utilizing this hybrid system encompass a range of capabilities including bioresorbable, colorimetric, gas-sensing, and LED fliers to address various future scenarios in remote sensing.

## Introduction

Wind-dispersed seeds play a crucial role in the survival of plant species by promoting widespread distribution of genetic material (1). To achieve this objective, many types of seeds incorporate distinctive passive flight mechanisms, with favorable aerodynamic features that include considerations in wing loading, weight distribution, and stability (2). Among the most well-known wind-dispersed seeds are those that parachute (e.g. dandelion seeds) or autorotate (e.g. maple seeds). Recent investigations unveil mechanisms responsible for generating lift in these fliers: dandelion seeds utilize a separated vortex ring (SVR) located above their center (3), whereas maple seeds rely on leading-edge vortices (LEVs) near their wing tip (4). These findings motivate fundamental studies that examine porosity (5), pappus angle (6), effects and control of flow instability (7) for dandelion seeds and that investigate Coriolis effects (8), scaling laws (9), and LEV circulation (10) for maple seeds and others.

Each mechanism presents distinct advantages and disadvantages; an autorotating seed can bear a heavy weight, resulting in a higher Reynolds number (Re=UD/v, the ratio between inertial and viscous forces, where *U* is the seed velocity, *D* is the seed diameter, and *v* is the kinematic viscosity of the surrounding fluid). *Re* lies in the range of 10^3^–10^4^ for maple seeds and 10^2^–10^3^ for dandelion seeds (3, 11). Dandelion seeds are susceptible to wind currents, characterized by a low Stokes number (a dimensionless number describing the behavior of particles in a fluid flow), making them an ideal choice as Lagrangian tracers (12). Thus, with a given size, the autorotating flight mechanism is preferred when the integration of a heavy payload is required. On the other hand, parachuting flight should be considered for fluid-related measurements such as assessing the anisotropic and inhomogeneous nature of atmospheric turbulence.

Recent efforts focus on the development of passive flier systems inspired by wind-dispersed seeds for potential applications in environmental monitoring and other contexts that require coverage of electronic components or functional materials across vast spatial scales (13). Examples range from battery-free wireless (14), light-driven (15, 16), biodegradable (17, 18), shape-morphing (19, 20), and power efficient (21) flier systems inspired by various wind-dispersed seeds including parachuting, autorotating, and gliding types. Contemporary advancements in soft electronic technologies create the possibility for integrating miniaturized electronic payloads conformal to nearly any 3D deformable surface (22), including curved wing areas with different shapes (13, 18).

Most aerodynamic studies examine the mechanics of existing wind-dispersed seeds or nonfunctional simple structures. Related topics in materials research center mainly on the fabrication of flight structures and functional payloads. Previous research features single passive flight mechanisms, based on established aerodynamic phenomena. The work presented here aims to bridge the gap between fundamental fluid mechanics and applied materials science, thereby establishing passive flight mechanisms that do not exist in nature. The approach encompasses a multidisciplinary framework that combines insights from biology, experimentation, analytical methods, and computational fluid mechanics as well as integrated manufacturing technologies capable of addressing applications in remote sensing.

Specifically, the work introduces a hybrid passive flier system, fabricated by a process in controlled buckling that simultaneously integrates SRV and LEV mechanisms. The studies employ advanced experimental techniques and computational simulations to understand the combined aerodynamics and flow physics comprehensively. An analytical framework for future extensions complements these empirical findings. Demonstrations with functional payloads illustrate optimal strategies and capabilities.

**Fig. 1.**
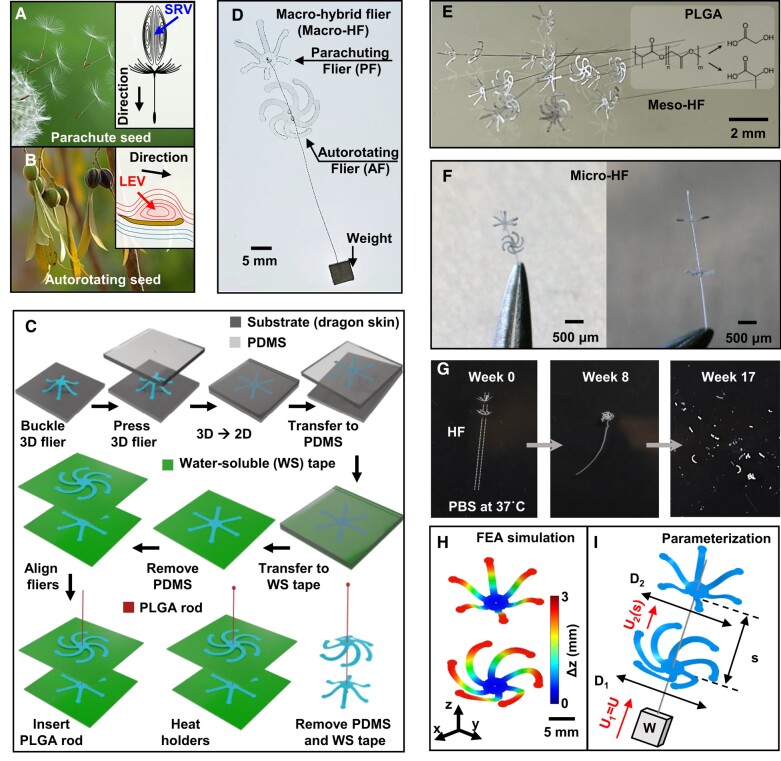
Hybrid fliers and associated fabrication schemes. Photographs of A) parachute (dandelion) and B) autorotating (*Gyrocarpus americanus*) seeds; (inset) schematic illustrations of the corresponding vortex dynamics. C) Procedures to fabricate a biodegradable HF. D) Photograph of a macro-HF, consisting of PF and AF, and an attached weight. E) Photograph of a meso-HF formed in PLGA (thickness ∼60 mm). F) Optical micrographs of a micro-HF at two different angled views. G) Images of dissolution of an HF at various times following immersion in PBS (pH 7.4) at 37 °C. H) FEA simulations of the geometry of an HF. I) Schematic diagram of an HF to define key parameters: diameters of bottom ($D_{1}$) and top fliers ($D_{2}$); corresponding incoming velocities ($U_{1}$ and $U_{2}$); separation (*s*) and weight (*W*).

The SVR corresponds to a detached ring of recirculating fluid formed by flow passing through the porous pappus (Fig. 1A). The leading-edge vortex (LEV) arises from flow separation at the leading edges autorotating wings, resulting in the development of a rolling shear layer along the surfaces of the wings (Fig. 1B). The porosity of the dandelion maximizes aerodynamic loading and minimizes material requirements (3). LEV increases the loading as well, but requires wing attachment, akin to certain insect mechanisms (23). The distinction between the two lies in the fact that (i) maple seeds operate at a high *Re* (24, 25), while (ii) dandelion seeds offer stability (7) due to their porous pappus and a low center of mass with respect to their geometrical center. The hybrid system introduced here harnesses the benefits and mitigates the drawbacks of these aerodynamic features.

## Results

The fabrication process of such a hybrid flier (HF) involves multiple, controlled mechanical buckling motions that occur perpendicular to surfaces of planar precursors using methods adapted from previous work (13) (Fig. 1C). This process forms 3D fliers of poly(lactic-co-glycolic acid) (PLGA) (see Materials and methods and Fig. S1). Pressing these fliers against a flat surface with an elastomeric slab (polydimethylsiloxane, PDMS) flattens them into planar shapes for transfer to a water-soluble (WS) tape. Aligning pairs of such flattened fliers using a microscope enables insertion of a PLGA rod through holes in the centers. Heating the rod at its tips ends forms a bead structure to prevent detachments of the fliers. Dissolving the WS tape by immersion in water causes the fliers stacked with two 2D precursors (2D–2D) to transform into hybrid fliers stacked with two 3D fliers (3D–3D). This scheme provides a scalable, reliable means for mass production, compatible with hybrid fliers of various sizes, from macro (cm scale; Fig. 1D) and meso (mm scale; Fig 1E) to micro (μm scale; Fig 1F). These hybrid fliers are both light weight (a typical macrohybrid flier weighs ∼10 mg) and bioresorbable (Fig. 1G). Finite element analysis (FEA) plays a crucial role in the design of hybrid fliers that combine parachuting and autorotating aerodynamic characteristics (Fig. 1H). The FEA predicts the 3D geometries that result from compressive buckling and helps to identify preferred 3D fliers for integration.

A shared configuration for the numbers and dimensions of wings in parachuting fliers (PF) with the straight wings and fraction of $\phi_{1}=0.28$ and autorotating fliers (AF) with the curved wings and fraction of $\phi_{2}=0.4$ allows comprehensive exploration of aerodynamic effects and associated flow physics, along with a concise analytical approach. The fraction, *ϕ*, is defined by $A_{\mathrm{eff}}/A$, where $A=\pi D^{2}/4$ is the cross-sectional area and $A_{\mathrm{eff}}$ is the effective area of flier's wings, when it is projected on 2D plane. Effects of (i) diameter ratio, $D_{2}/D_{1}$, where $D_{1}$ and $D_{2}$ are diameters of AF and PF, respectively, (ii) separation distance, *s*, and (iii) resulting velocity conditions of $U_{1}=U$ and $U_{2}(s)$, where *U*, $U_{1}$, and $U_{2}$ are free-fall, incoming velocities of autorotating, and PF, respectively, are considered. These parameters underpin a systematic approach to aerodynamic modeling (Fig. 1I).

**Fig. 2.**
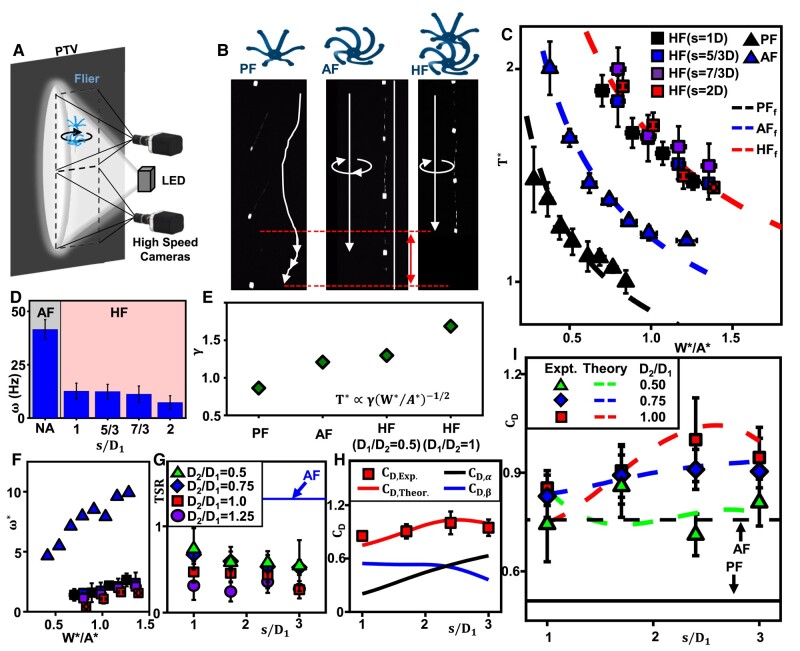
Aerodynamics of hybrid fliers. A) Schematic illustration of the PTV setup. B) Optical images of a free-falling (left) PF, (middle) AF, and (right) HF. C) Normalized descent time ($T^{*}$) vs. normalized wing loading ($W^{*}/A^{*}$) of a PF, AF, and HF via PTV; corresponding inverse squared relations $T^{*}\propto \gamma /\sqrt{W^{*}/A^{*}}$ (dashed lines); *γ* is the descent factor. D) Rotational speed (*ω*) between an AF and HF as a function of normalized separation (s/D) via PTV. E) *γ* of a PF, AF, and HF. F) Normalized rotational speed ($\omega^{*}$) vs. $W^{*}/A^{*}$ of an AF (triangle) and HF (square symbols) via PTV. G) TSR of an AF (solid line), and an HF with various $D_{2}/D_{1}$ and s/D (symbols) via PTV. H) Analytical solutions for $C_{D,\alpha }$, $C_{D,\beta }$, and $C_{D}$ of an HF with $D_{2}/D_{1}$ = 1 as a function of s/D (solid lines), overlaid by the corresponding experimental results (square symbols). I) Comparison between the analytical (solid lines) and experimental (symbols) results with various $D_{2}/D_{1}$ and s/D. The error bars indicate the standard deviation of three samples (*n* = 3) for every s/D and $D_{2}/D_{1}$ configurations.

More than 300 experimental tests involving particle tracking velocimetry (PTV) (see Fig. 2A and Materials and methods) (26) serve to unravel distinct aerodynamic characteristics of the HF in comparison to both PF and AF. These experiments explore the influence of $D_{2}/D_{1}=[0.5,\;1.25]$, $s/D_{1}=[1,3]$, and weight, W=[0.145,0.574]mN mN, where $D_{1}=1.78\;\mathrm{cm}$. The configuration of HF, with AF positioned beneath, remains constant throughout the study due to the reliance of the AF on a uniform flow for autorotation (Video S1). As expected, the PF exhibits the fastest rate of falling characterized by chaotic behaviors, attributed to its weight and resulting *Re* exceeding its effective range (>1,000). In contrast, the AF demonstrates a slower and more stable falling dynamics, with a rotational speed (*ω*) of ∼40Hz. The HF exhibits the slowest terminal velocity and rotational speeds below 20 Hz during free fall due to factors such as friction and moment of inertia (Fig. 2B and D). A rapid rotation speed is not always ideal, as it results in insufficient time for the full development of LEV while attached to the wing (18).

Dimensionless analysis involving the descent time, $T^{*}=T/T_{max}$ and rotational speed $\omega^{*}=\omega /gT$ as a function of the normalized wing loading $W^{*}/A^{*}=WA_{P}/W_{wf}A$ capture their overall aerodynamic performance and indicate their potential for sensor integration (Fig. 2C and F). $T_{\max}$ is the maximum observed descent time (PF with 0.533 mN), *g* is the gravitational force, $W_{wf}$ is the payload weight. $A_{P}=\frac{\pi \phi_{1}D_{1}^{2}+\pi \phi_{2}D_{2}^{2}}{4}$ represent the physical areas of the wings of a flier, respectively. The normalized wing loading $W^{*}/A^{*}$ is defined as the ratio between the wing loading with total weight and cross-sectional reference area, W/A, and with payload weight and physical area of the wings $W_{wf}/A_{P}$. Compared to the rotation of the AF, the HF exhibits significant enhancements in its normalized descent factor, $\gamma =T^{*}\sqrt{W^{*}/A^{*}}$, showing improvements of 100 and 40% relative to its PF and AF counterparts (Fig. 2C and E). Furthermore, within the HF systems, the HF exhibits a substantial reduction (>75%) in the dimensionless rotational speed $\omega^{*}$ (Fig. 2F). A comparison of *T* and W/A (Fig. S2 left) and drag coefficient, $C_{D}=2W/\rho_{f}U^{2}A$, where $\rho_{f}$ denotes the fluid density, facilitates studies of the aerodynamic performance. The results indicate that the hybrid system retains the aerodynamic advantages of both the LEV and SRV while securing additional space for mounting sensors or other components or indicators.

**Fig. 3.**
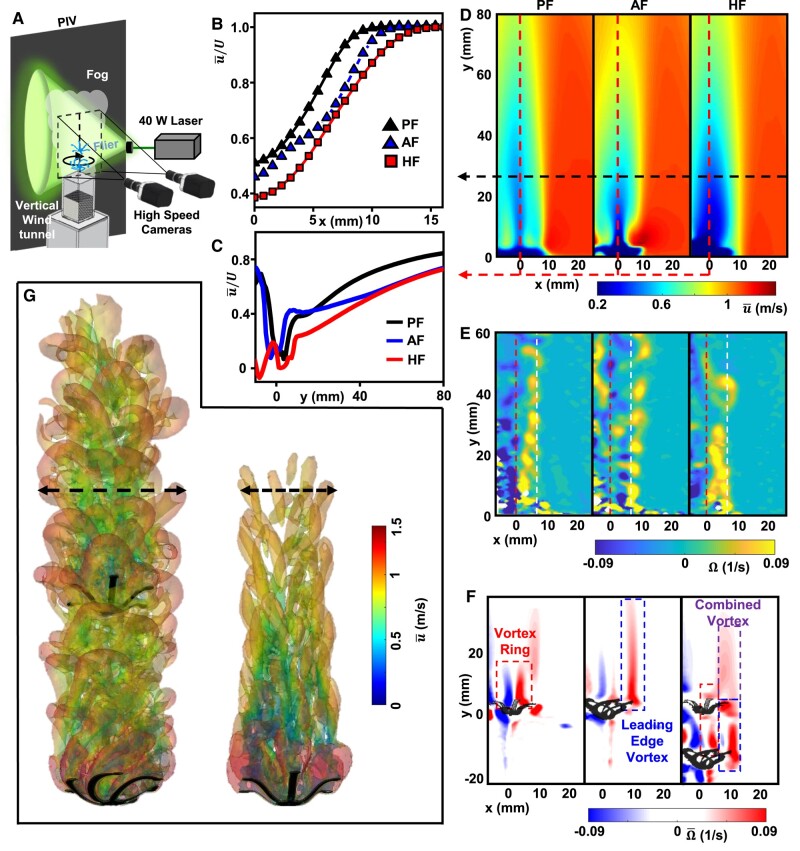
Flow physics of hybrid fliers. A) Schematic illustration of the PIV and vertical wind tunnel setup. Velocity profiles of a PF, AF, and HF along B) the spanwise direction at $y=1.5D_{1}$ and C) streamwise direction at x=0 via PIV. D) Mean velocity ($\bar{u}$; horizontal and vertical dashed lines indicate the spanwise and streamwise locations for velocity profiles, respectively), E) Instantaneous vorticity (Ω; left and right dashed lines indicate the center axes of the SVR and LEVs), and (F) average vorticity ($\bar{\Omega }$; dashed boxes indicate the effective areas of SVR, LEV, and combined vortex) fields via PIV. G) 3D visualization of vorticity (Q-criteria) in the wake colored by air speed; (left) HF and (right) PF via computational fluid dynamics; dashed lines indicate the spanwise length of the wake.

An analytical methodology establishes a theoretical framework for $C_{D}$ of these systems. $C_{D}$ associated with HF, with the assumption of a linear addition, can be derived by considering the drag force exerted on each flier component as follows:


(1) $$C_{D}=\frac{C_{D,1}U_{1}^{2}A_{1}+C_{D,2}U_{2}^{2}A_{2}}{U^{2}A},$$

where subscripts 1 and 2 represent quantities associated with AF and PF. The AF is positioned in the upwind location, so *U* is representative of $U_{1}$. However, $U_{2}$ for the PF is influenced by the wake generated by the AF and varies based on the separation distance, *s*, between the two fliers. The wake center velocity distribution of a single PF is obtained through particle image velocimetry (PIV) experiments (see Fig. 3C) to estimate $U_{2}(s)$. It is approximated as *α* times the wake center velocity at y=s by considering the Gaussian distribution, as evident in Fig. 3B for the velocity deficit in the wake, where the *y*-axis is the streamwise direction, and the center of the AF is placed at the origin. In the 2D simulations, a satisfactory estimation of $U_{2}$ was achieved with α=1.36 for separations s/D≤6. This estimation can be validated by comparing the drag force on the downwind flier with the wake center velocity of a single flier (see Fig. S2B). When the ratio of $D_{1}$ to $D_{2}$ is equal to 1 ($D_{2}/D_{1}=1$), the drag coefficient $C_{D}$ of the HF can be expressed as $C_{D}=C_{D,1}^{*}+C_{D,2}{\left(\frac{\alpha U_{C}(s)}{U}\right)}^{2}$ with α=1.36, where $C_{D,1}^{*}\;=\;0.9C_{D,1}$, which is assumed to be a constant independent to the separation distance (27) (Fig. S3). However, it is important to note that the tip speed ratio (TSR) for every HF is lower than that of the single AF (Fig. 2G). The TSR corresponds to the ratio between the speed of the rotating wing tip and the free-fall velocity. In wind turbine systems, it is known that a higher TSR within a broad range leads to a larger velocity deficit. The thrust coefficient exhibits a linear relationship with TSR at comparatively small values (≤3). The thrust corresponds to the drag and a linear term is added as a function of TSR, *β* TSR(s) + *Υ*, which reflects the rotation effect. Accounting for TSR (28) and $D_{2}/D_{1}$, Eq. 1 can be further expressed as follows:


(2) $$C_{D}=C_{D,1}^{*}+\left[\beta \;\mathrm{TSR}\left(s,\frac{D_{2}}{D_{1}}\right)+\Upsilon \right]+\left[C_{D,2}{\left(\frac{\alpha \;U_{C}(s)}{U}\right)}^{2}{\left(\frac{D_{2}}{D_{1}}\right)}^{2}\right].$$

The analytical solution of $C_{D}$ agrees with experimental results as a function of *s* (Fig. 2H) and $D_{2}/D_{1}$ (Fig. 2I) using the empirical coefficient β=0.47 and Υ=−0.61, where $C_{D,\alpha }=C_{D,1}^{*}+\left[\beta \;\mathrm{TSR}\left(s,\frac{D_{2}}{D_{1}}\right)+\Upsilon \right]$ and $C_{D,\beta }=\left[C_{D,2}{\left(\frac{\alpha \;U_{C}(s)}{U}\right)}^{2}{\left(\frac{D_{2}}{D_{1}}\right)}^{2}\right]$, thereby revealing the associated physics. It is worth pointing out that when the parachuting flier is larger than the autorotating flier (e.g. $D_{2}/D_{1}=1.25$), the analytical solution begins to deviate from the experimental data (Fig. S2C). The deviation is attributed to the fact that when the downwind flier is larger, it is subject to a high-momentum fluid flow diverted by the upwind flier, leading to increased drag at smaller s/D ratios.

PIV experiments conducted under controlled conditions emulate incoming velocities of a HF with $D_{2}/D_{1}=S/D=1$ within the range of *U*. The experiments use a custom vertical wind tunnel setup (Fig. 3A and Video S2) suitable for 3D numerical simulations (see details in the Materials and methods section and Fig. S4). This combined experimental and computational approach serves to validate the unique aerodynamic traits of the fliers and the underlying flow physics, along with the interactions of governing vortex dynamics. Mean velocity profiles in the spanwise (Fig. 3B) and streamwise (Fig. 3C) directions along with the corresponding flow fields, generated by AF, PF and HF (Fig. 3D) highlight that the momentum deficit of the HF flier is the highest in both directions, resulting in the highest overall $C_{D}$. Similar to $C_{D}$ from free-fall studies, $C_{\mathrm{DMD}}$, estimated by the momentum deficit, of the HF, where $C_{\mathrm{DMD}}=\frac{2D_{1}}{AU^{2}}\int_{x=0}^{x=1.5D_{1}}\bar{u}(U-\bar{u})dx$ exhibits ∼56 and ∼15% improvements compared to PFs and AFs, respectively. Slightly lower improvements than $C_{D}$ are due to different incoming velocity conditions corresponding to each flier, not fully capturing the coupled mechanics between structures and fluid flow. Instantaneous (Fig. 3E) and average (Fig. 3F) vortex fields further indicate additive characteristics of $C_{D}$ from both systems. The AF and PF demonstrate periodic vortex sheddings, but at different spanwise locations of the flier; LEV occurs near the wing tip of the AF and SRV occurs in the center of the PF (Fig. 3E), allowing for the coexistence and merging of both vortex structures along the spanwise direction (Fig. 3F). It is worth noting that vortex breakdown can induce aerodynamic torque, leading to flier oscillations, which may eventually result in $C_{D}$ fluctuations. For the numerical approach, an arbitrary Lagrangian–Eulerian (ALE) technique with variational multiscale (VMS) formulation solves the Navier–Stokes equations on a moving spatial domain of flier systems (29, 30). Figure 3G suggests that velocity fluctuations may affect the drag coefficient through various mechanisms, such as altering flow structure, increasing turbulence, and triggering flow instability. Indeed, the flow deviates significantly from the Stokes-like regime, indicating that flow instability is a contributing factor (see Note S1 and Video S3 for additional details).

**Fig. 4.**
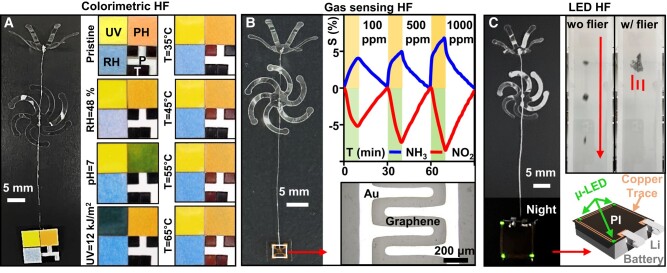
Functional and improved hybrid fliers. A) Photograph of a colorimetric HF (left) and resulting color changes for RH, pH, UV exposure, and temperature (right). B) Photograph of a gas-sensing HF (left), measurements of NH_3_ and NO_2_ (right top), and microscope image of the sensor (right bottom). C) Photograph of an LED HF (left), wind tunnel experiment without (top middle), and with the flier (top right), as well as illustration of the LED payload.

The fabrication scheme provides versatile options for environmental and atmospheric sensing applications. Figures 4A and S7 show colorimetric information related to humidity (S-8028, ULINE), pH (Cat#93, Hydrion), UV exposure dose (N010-002, Con-Trol-Cure), and temperature (EW-09035-51, Digi-Sense), each extracted from a colorimetric HF. Figures 4B and S8 demonstrate the possibility of a gas-sensing HF by integrating a chemiresistive-type gas sensor (31, 32) for two common polluting gases, NH_3_ and NO_2_. Based on the p-type semiconductor properties of graphene as a sensing material, the resistance of the sensor increases when NH_3_ adsorbs on the graphene, inducing a charge transfer, and decreases upon exposure to NO_2_ (33). The sensor responds from relatively low (<100 ppm) to high (up to 1,000 ppm) concentrations of the gases with full recovery and correlated linear responses. Figures 4C and S9 demonstrate a HF that integrates miniaturized light-emitting diodes (LED), often used in flexible optoelectronic systems (34, 35), along with a lithium-ion battery (350 mg), designed for optical tracking of the flier's full kinematics including the angle of rotation and 3D translation during a nocturnal period. Video S4 highlights the levitation of a heavy LED payload attached to the HF under the condition of a balanced incoming flow from a vertical wind tunnel (see Materials and methods for specific details of functional hybrid fliers).

**Fig. 5.**
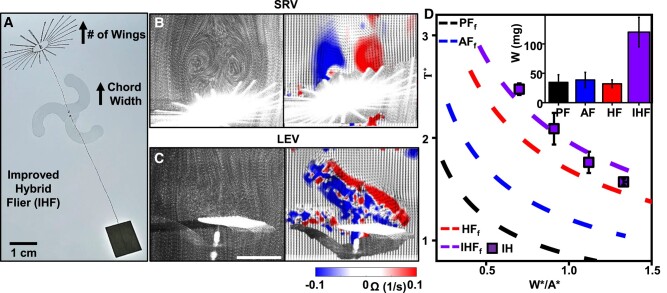
Improved hybrid fliers. A) Photograph of an IHF. Superimposed images of the flow visualization above the improved PF B) and improved AF C) as well as corresponding vorticity fields (right). D) $T^{*}$ vs. $W^{*}/A^{*}$ of IHF (square) via PTV; corresponding inverse squared relations $T^{*}\propto \gamma /\sqrt{W^{*}/A^{*}}$ (dashed lines); average weights of payloads tested for PF, AF, HF, and IHF via PTV (inset).

Investigations of the aerodynamics and flow physics of HF can extend beyond a singular design configuration of individual fliers. With emphasis placed on the influences of diameter ratio and separation, the enhancements to behaviors of individual fliers can be integrated into the analytical framework presented here, with the potential to further augment the collective performance of the HF. Figure 5A shows an example of an improved hybrid flier, IHF, that follows from increasing and decreasing the number of wings of the PF and AF, respectively, while fixing the overall physical area. The objective is to foster sustainable and high levels of SRV and LEV by promoting a homogeneous porosity and large chord width, respectively. PIV experiments on the near field above each flier illustrate and quantify SRV (Fig. 5B) and LEV (Fig. 5C). $T^{*}$ vs. $W^{*}/A^{*}$ of IHF demonstrates an additional 16% improvement of *γ* to HFs (127 and 62% compared to PFs and AFs), opening many possibilities for such a system (Figs. 5D and S10).

## Discussion

The concepts reported here lead to enhanced performance in passive flying systems of relevance to remote sensing. The key advances are in (i) complex, biodegradable hybrid 3D structures in designs that consider flow physics and aerodynamics, (ii) schemes to exploit two distinct passive flying mechanisms inspired by wind-dispersed seeds, i.e. leading-edge and separated ring vortices, (iii) comprehensive insights derived from a series of detailed experiments and simulations that elucidate the key physics and enable the development of a concise analytical framework, (iv) application of this analytical framework to various hybrid systems, and (v) platforms that can accommodate significantly larger and heavier payloads compared to those of previous studies (13, 18), for aerial dispersal of advanced device technologies. Although not explicitly investigated in this work, the remote sensing applications of these heterogeneous 3D architectures may benefit from recently reported biomedical devices (36–38), metamaterial structures (39, 40), and electronic platforms (41, 42). Various categories of relatively heavy components including antennas, microcontrollers, sensors, and miniaturized batteries can be integrated easily. These same technologies may facilitate additional fundamental studies in fluid mechanics and flight dynamics under various conditions. Such research may reveal other unusual passive flying mechanisms and their associated key features. For example, investigating the fluid–structure interaction within an array of hybrid systems and in regard to flight instability and exploring hybrid systems comprising additional passive flying mechanisms such as gliding and fluttering wind-dispersed seeds hold promising prospects for future research.

## Materials and methods

### Fabrication and measurement of macro-, meso-, and micro-bioresorbable hybrid fliers

Various autorotating, parachuting and hybrid fliers can be efficiently produced using bioresorbable PLGA. PLGA is environmentally benign, and it is naturally biodegradable via hydrolysis into its constituent monomers, lactic acid and glycolic acid, under mild environmental conditions (∼300 K). Fabrication of 2D precursors in PLGA began with laser ablation to define shape selected based on FEA-modeling of the buckling process, from thin PLGA films (thickness of ∼60 μm). Transferring 2D structures onto a PDMS elastomeric substrate allowed initiation of a mechanically guided assembly process that transformed the 2D precursors to 3D structures. Heating to 70 °C for 1 min in an oven relaxed the strains in the PLGA and fixed the 3D geometry. Autorotating and parachuting 3D fliers were transferred to a WS tape and combined as hybrid fliers by inserting a PLGA rod through center holes formed in the fliers. Removing WS tapes induced reconstruction of the 3D structures to create hybrid fliers. Fig. 1E shows the buckling approach to generating bioresorbable microsale hybrid fliers, as observed using digital optical microscope (Keyence). 3D buckling offers advantages over conventional fabrication methods, including the ability to accommodate a wide range of types, sizes, and structural features within the fliers, in a manner compatible with any material or microsystem technology in planar form. This flexibility enables the design and fabrication of intricate and complex fliers with functionalities, improved performance capabilities, and adaptability to specific application requirements. Biodegradation tests provided insights into the environmental impact and lifespan of the hybrid fliers. The test involved monitoring the degradation progress in Phosphate Buffered Saline (PBS) (at 37 °C) over time (Fig. 2F). At week 0, the flier exhibited no visible damage, indicating its initial integrity. At week 8, significant changes were evident. The rod in the flier underwent deformation, exhibiting bending and discoloration. At week 17, the flier disappeared, corresponding to complete biodegradation.

### Finite element analysis

Nonlinear postbuckling behaviors of the 2D precursor structures were evaluated using 3D FEA in the commercial software package Abaqus. The simulations involved modeling the PLGA material as linear elastic, with an elastic modulus of 1.37 GPa and Poisson’s ratio of 0.44. Four-node shell elements were selected to mesh the 2D precursor structures. To ensure computational accuracy, mesh size convergence was tested. Overall, the deformed 3D configurations and strain distributions were obtained for different levels of compression in the 2D precursor structures.

### PTV for aerodynamic measurements

Free-fall experiments using PTV were recorded by two synchronized 2MP Emergent HT-2000M at 450 fps covering the investigation area of 0.12 × 0.5 m^2^ (*W*  *×H*) illuminated by a continuous LED light source. After removing the background image, raw images were binarized. The flier velocities were estimated using u=ΔL/Δt, where ΔL is the displacement of the rectangular artificial weight attached below the HF (Fig. 1D) between successive frames, and Δt=1/450 s is the inverse of the PTV sampling frequency. The rotation speed of the flier is estimated by doubling the period of the variation in the projection area of the rectangular-shaped artificial weight. Preprocessing, calibration, 3D reconstruction, tracking, and postprocessing exploited 3D-PTV codes described previously (43).

### Preliminary 2D simulations

Planar numerical simulations were employed to augment the analytical model for the drag coefficient of the HF. These simulations provided an estimation of the velocity distribution and drag force on individual fliers, as well as HF configurations with separations between two fliers (*s*/*D*) ranging from 1 to 6, where *D* represents the diameter of the flier. Only hybrid fliers of equal size (*D* = 10 mm) were considered for these simulations. The computational domain encompassed a region 25*D* in the streamwise direction and 10*D* in the spanwise direction. The upwind flier was positioned at a distance of 10*D* from the inlet. The computational domain was discretized into elements with a size of 0.08*D*. Additionally, a finer mesh of ∼0.008*D* was employed close to the flier surface. The ambient fluid was specified as air at a temperature of 20 °C, and the inlet velocity was set at $U_{\infty }$ = 1 m/s. The unsteady Reynolds-averaged Navier–Stokes equations with the *k*–ω SST model were utilized for the simulations. A timestep of 1 ms was employed, and the computations were carried out over a duration of 3,000 timesteps.

### PIV for wake characterizations

PIV experiments used two high-speed cameras (HT-2000M, Emergent) and a synchronized high-speed laser (527-40-m, Terra) above a custom vertical windtunnel, setup similar to previous work (13). The measurements defined the wake and vortex dynamics of fixed autorotating, parachuting and hybrid fliers in an investigation area of 60 × 130 mm^2^ (*W*  *×H*). Fliers were exposed to flow velocities similar to those associated with free-falling velocities measured by PTV experiments. Green, dyed water droplets generated from a nebulizer served as tracer particles. The PIV experiments were recorded at 1,000 Hz and processed by the open-source code, PIV lab (44). Over 6,000 image pairs were processed using the Fast Fourier Transform (FFT) window deformation method with 3 passes of interrogation areas from 64 × 64 to 16 × 16 pixels with 50% overlap, resulting in over 14,000 velocity vectors and Δ*x*=Δ*y* = 0.769 mm.

### Computational fluid dynamics simulations

The aerodynamics of the fliers are modeled by using Navier–Stokes equations of incompressible flows. An ALE technique is applied to impose the Navier–Stokes equations on a moving spatial domain to handle the flier motion. The governing equations are given as


$$r_{M}(u,p)\;:=\rho \frac{\partial u}{\partial t}+\rho (u-\hat{u})\cdot \nabla u-\rho b-\nabla \cdot \sigma =0$$

$$r_{C}(u):=\;\nabla \cdot u=0$$

where $u=(u_{1},u_{2},u_{3}{)}^{T}$ denotes the velocity vector, $\hat{u}$ denotes the mesh velocity vector, σ=−pI+2με(u) is the Cauchy stress tensor, *p* denotes the pressure, ρ denotes the density of the fluid, *μ* denotes the dynamic viscosity, and *b* is the body force per unit mass, $\nabla =(\partial /\partial x_{1},\partial /\partial x_{2},\partial /\partial x_{3}{)}^{T}$ is the gradient operator, I is the identity matrix, $\varepsilon (u)=\frac{1}{2}(\nabla u+\nabla u^{T})$ denotes the symmetric gradient of velocity field u. The Navier–Stokes equations are solved by using the VMS formulation which is acting as a large eddy simulation model for turbulent flows. A weak enforcement of essential boundary conditions acts as a near wall model. The combination of ALE and VMS has been extensively used to model numerous challenging flow problems (45–47). The ALE–VMS formulation is integrated in time using the generalized- α method (48). The equations are linearized using the Newton–Raphson method, and the resulting linear system is then solved using the generalized minimal residual method (49).

### Fabrication and measurement of the colorimetric HF

Colorimetric indicators for pH, relative humidity (RH), temperature, and UV sensing were fabricated from pH test paper (Cat#93, Hydrion), humidity indicator (S-8028, ULINE), reversible temperature label (EW-09035–51, Digi-Sense), and UV fastcheck strips (N010-002, Con-Trol-Cure). Fabrication of colorimetric indicators began with laser ablation to define the desired dimensions and shapes. The colorimetric indicator was placed on a hot plate (Guardian 7000 Ceramic Heating and Stirring, OHAUS) for temperature calibration against measurements performed with an infrared thermometer (Lasergrip 1080, Etekcity). UV lamps (UVP Blak-Ray B-100A, Analytik Jena US) provided light with a 365 nm wavelength for exposing the indicator for 140 s. The UV intensity was measured as 8 mW at the same spot using a Power and Energy Meter (PM 200, Thorlabs) and a photodiode (S120VC, Thorlabs). One drop of pH 7 buffer solution was applied to the pH indicator to provide a neutral environment for pH sensing. For relative humidity measurement, the colorimetric indicator was placed in an ambient environment, and the RH was measured using a digital humidity thermometer (HT-86, Walfront).

### Fabrication and measurement of the gas-sensing HF

Monolayer graphene on colorless polyimide film was purchased from MCK Tech (Daejeon, Korea). The sample was washed with acetone and isopropyl alcohol and dried. To fabricate the electrodes of the sensor on the graphene, the sample was covered with a metal stencil mask having a channel interval of 200 μm, and Au and Cr were deposited, 40 and 1 nm, respectively, using a thermal evaporation technique. The sensor was attached at the bottom of the flier using an adhesive. The gas responses of the HF with the gas sensor were measured in a customized gas chamber system (Micro Probe system, MPS-CHH8C, NEXTRON, KOREA) at room temperature. The resistance was measured by reading through a microprobe of the gas chamber system between the electrode of the sensor and the gold electrode of the sample stage. The gas flow rate was maintained at 1,000 sccm by a mass flow controller (AFC 600, ATOVAC, Korea). NH_3_ and NO_2_ gas flows with different concentrations (100, 500, and 1,000 ppm) were prepared by diluting the gas with air. The real-time resistance was measured by switching the target gas (response time of 10 min) and air (recovery time of 20 min) under an applied voltage of 1 V using Keithley 2612B instrument. The sensitivity of the sensor was evaluated as follows:


$$\mathrm{Sensitivity}\;(\text{\%})=\frac{R_{a}-R_{0}}{R_{0}}\times 100,$$

where *R*_0_ is the initial resistance and *R_a_* is the chanced resistance after graphene reacted with the target gas.

### Fabrication and measurement of the LED HF

Pyralux AP8535R served as a substrate for the circuit. The top copper layer (17.5 μm thick) and traces for the LED (0.65 mm × 0.35 mm × 0.2 mm) were structured via direct laser ablation (LPKF U4). Hot-air soldering using low-temperature solder 637 (Indium Corp., Clinton, NY) bonded LEDs (Green 571 nm LED, Digi-Key Electronics, MN) to the respective pads. An ultralow-weight lithium-ion battery (0.33 g, 3 mm × 9 mm × 10 mm, PowerStream Technology, UT) supplied power to the LED.

## Supplementary Material

pgae110_Supplementary_Data

## Data Availability

All data in this study are included in the article and in the Supplementary material.
