# Supplementary material for: Functional bio-inspired hybrid fliers with separated ring and leading edge vortices
Source: PNAS Nexus. 2024 Mar 11;3(3):pgae110. doi: 10.1093/pnasnexus/pgae110 (PMC10957237; doi:10.1093/pnasnexus/pgae110)
Supplement: pgae110_Supplementary_Data [file pgae110_supplementary_data.zip › PNASNEXUS-PNASNEXUS-2023-01179R-s01.pdf]

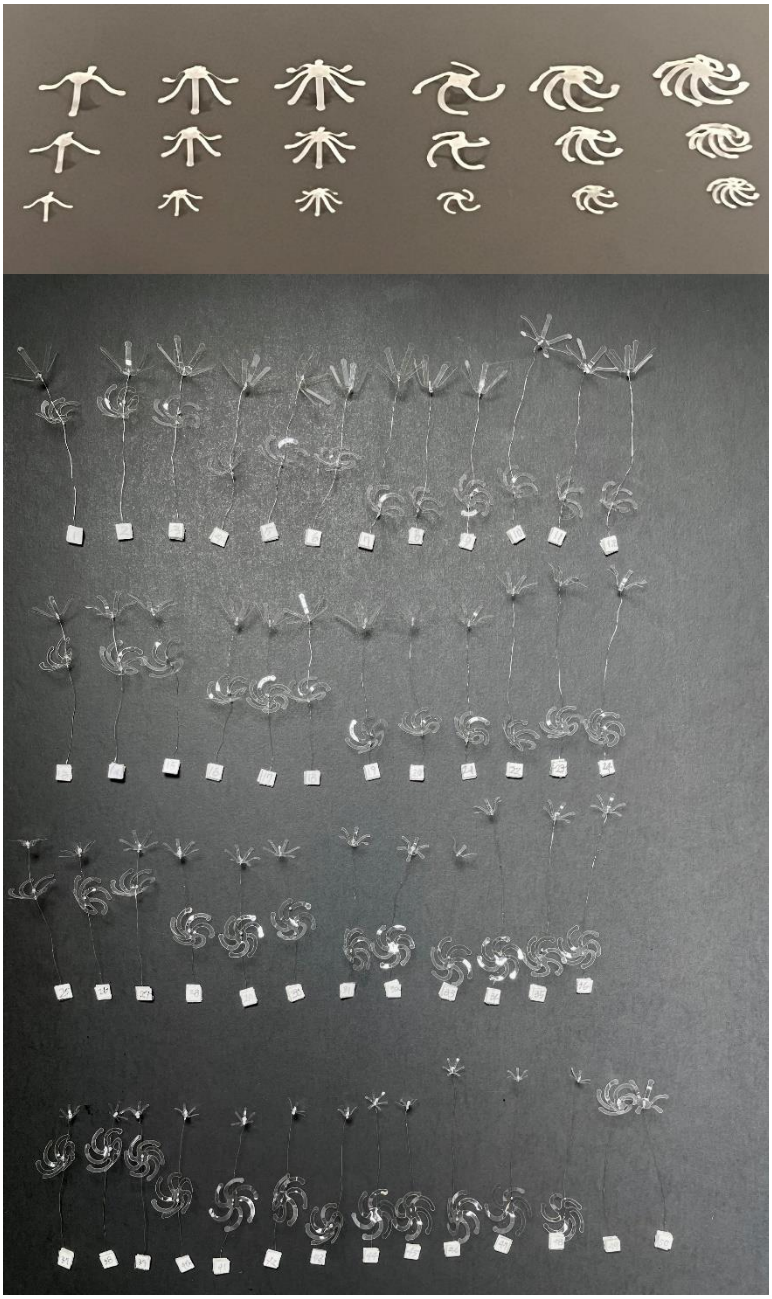

Supplementary Figure 1. Fabrication of hybrid fliers with various diameter ratios and separation distances.

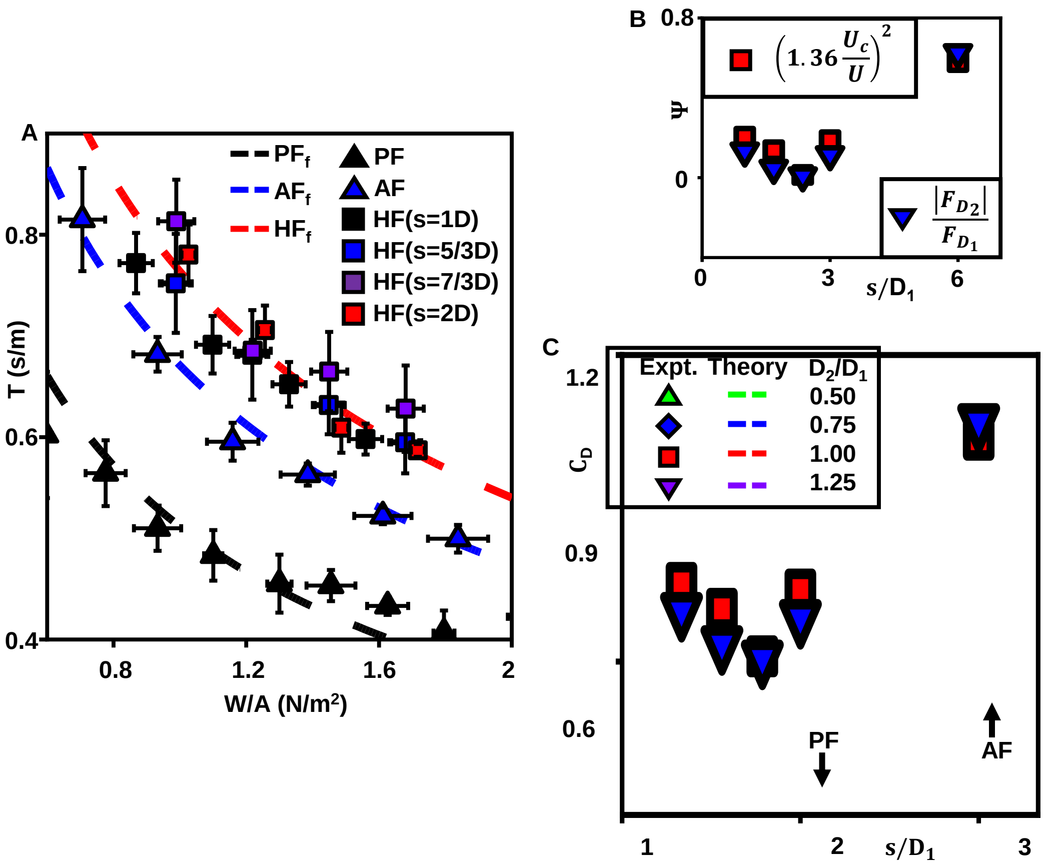

Supplementary Figure 2. (A) descent time (T) vs wing loading (W/A) of PF (black triangle), AF (blue triangle) and HF (square symbols) via PTV; corresponding inverse squared relations $T \propto \gamma/\sqrt{W/A}$ (dashed lines); γ is the descent factor. **(B)** Comparison between the normalized incoming velocity squared $((\alpha U_C(s))/U)^{\wedge 2}$ and the normalized drag force $|F_{D_2}|/F_{D_1}$ with respect to s/D_1 via 2D simulation. **(C)** Comparison between the analytical (solid lines) and experimental (symbols) results with various D_2/D_1 and s/D_1 including D_2/D_1=1.25.

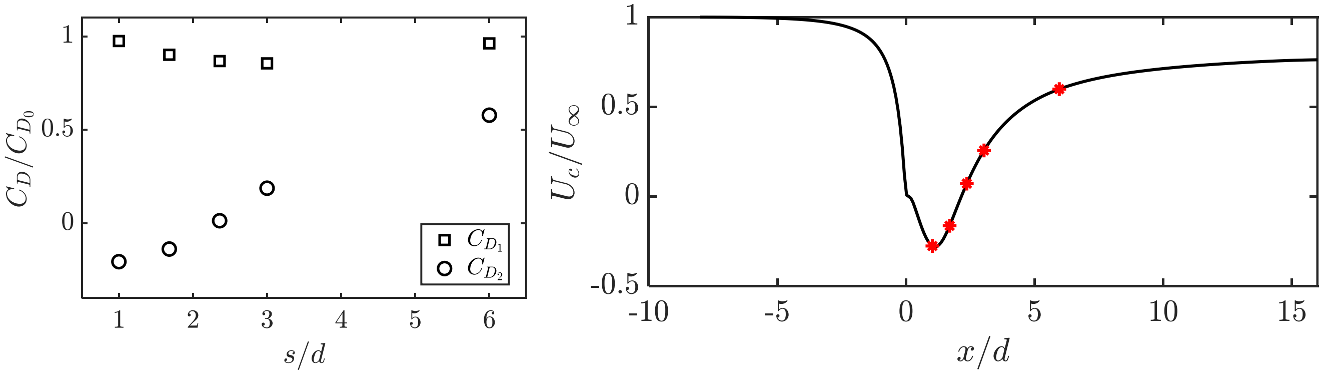

Supplementary Figure 3. Preliminary 2D simulations of drag coefficients for bottom and top fliers (left) and centerline velocity of the bottom flier with red dots marked at x/d = 1, 1.7, 2.4, and 3, which are the separation distances between two fliers considered in this study (right).

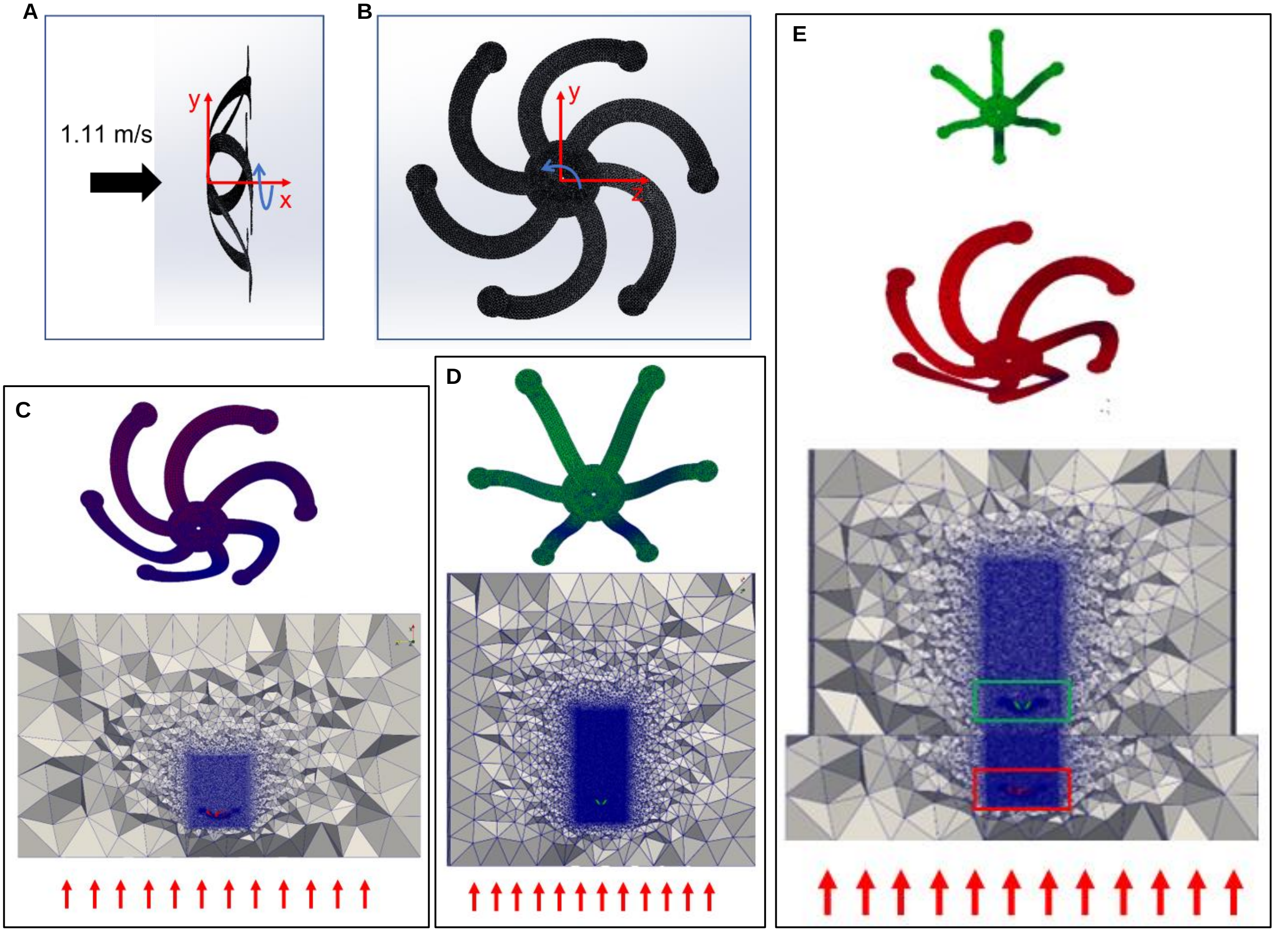

Supplementary Figure 4. CFD simulation setup and mesh details; **(A)** inflow conditions and **(B)** AF setup. Mesh details for **(C)** AF, **(D)** PF, and **(E)** HF simulations

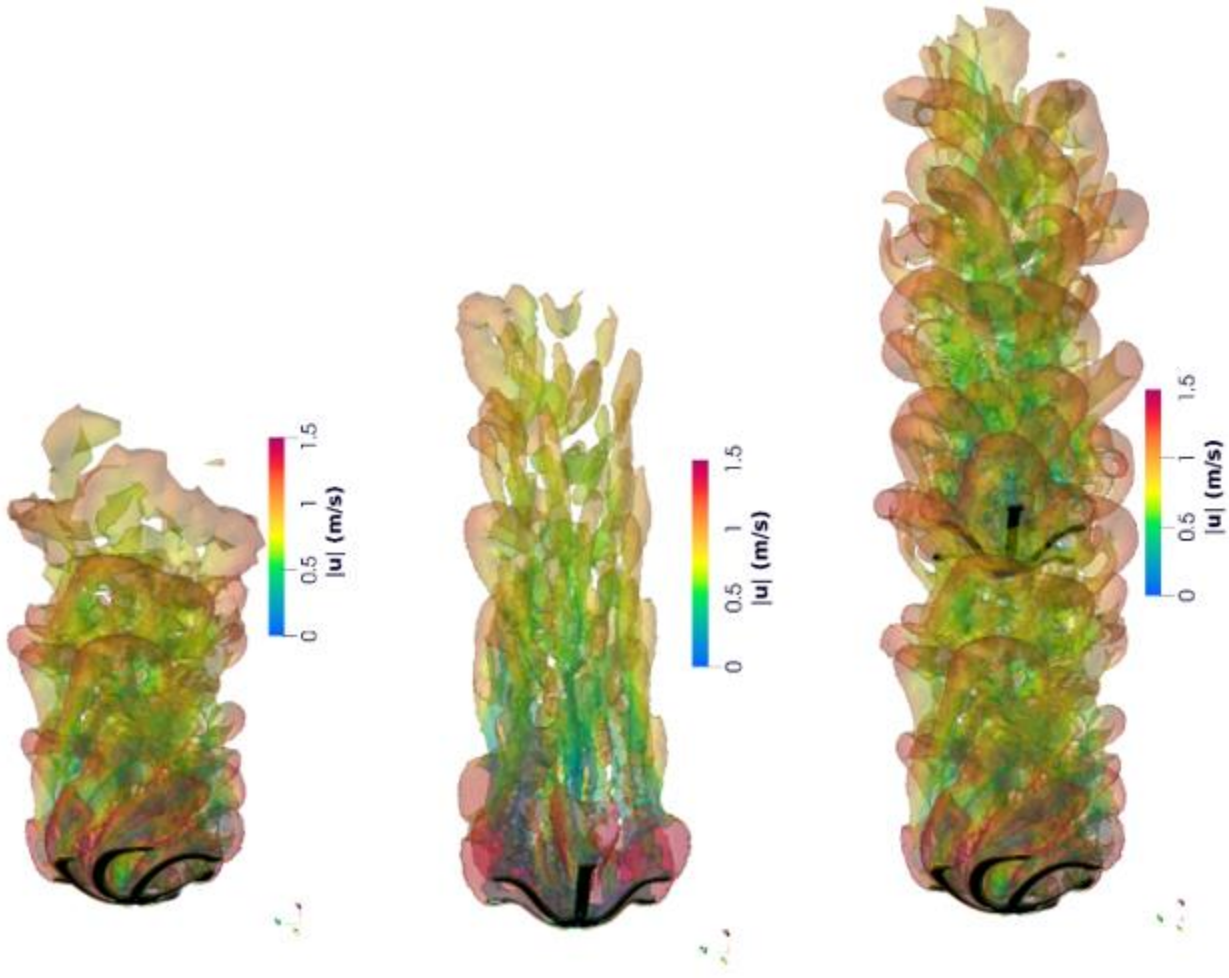

Supplementary Figure 5. Turbulent wake behind fliers. 3D visualization of vorticity (Q-criteria) in the wake colored by air speed; (left) AF, (middle) PF, and (right) HF via CFD

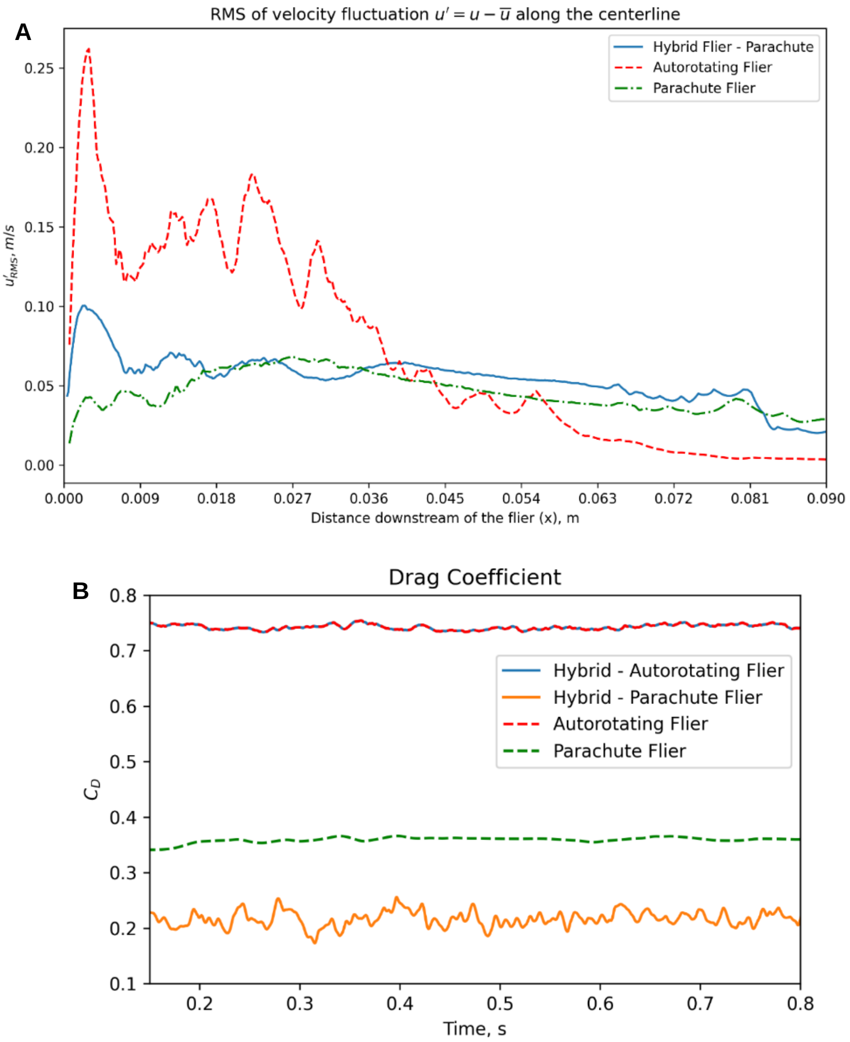

Supplementary Figure 6. (A) Root mean square of the velocity field fluctuation on the centerline along the downstream of the AF, PF, and HF. **(B)** Drag coefficients for flow past AF, PF, and HF.

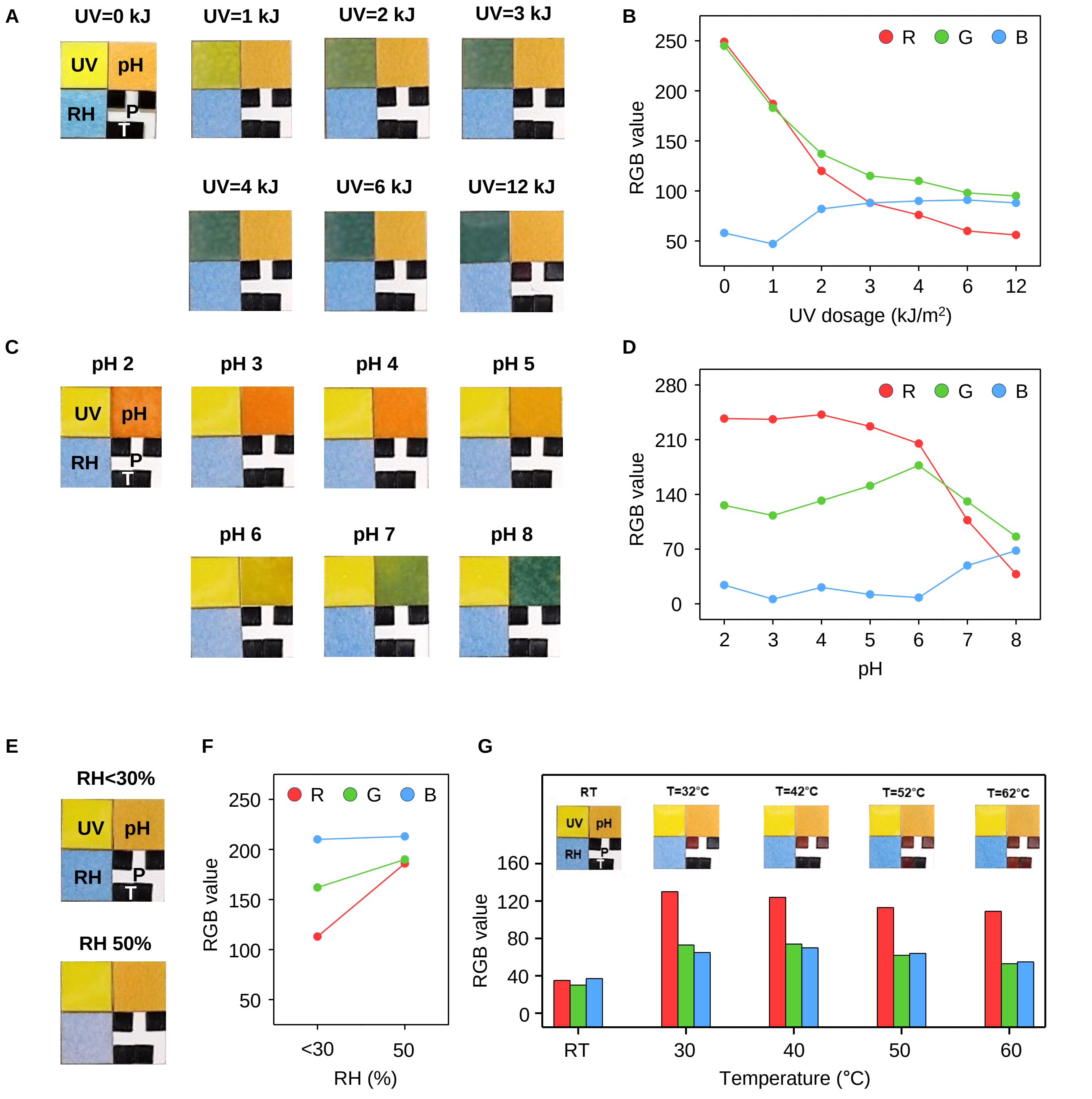

Supplementary Figure 7. Commercial colorimetric assays and quantitative analysis, extracting RGB values, for **(A, B)** UV and response to UVA up to 12 kJ/m^2, **(C, D)** pH and response to pH buffer solutions (pH 2-8), **(E, F)** RH and response to RH 50%, **(G)** Temperature and response to 32 to 62 °C.

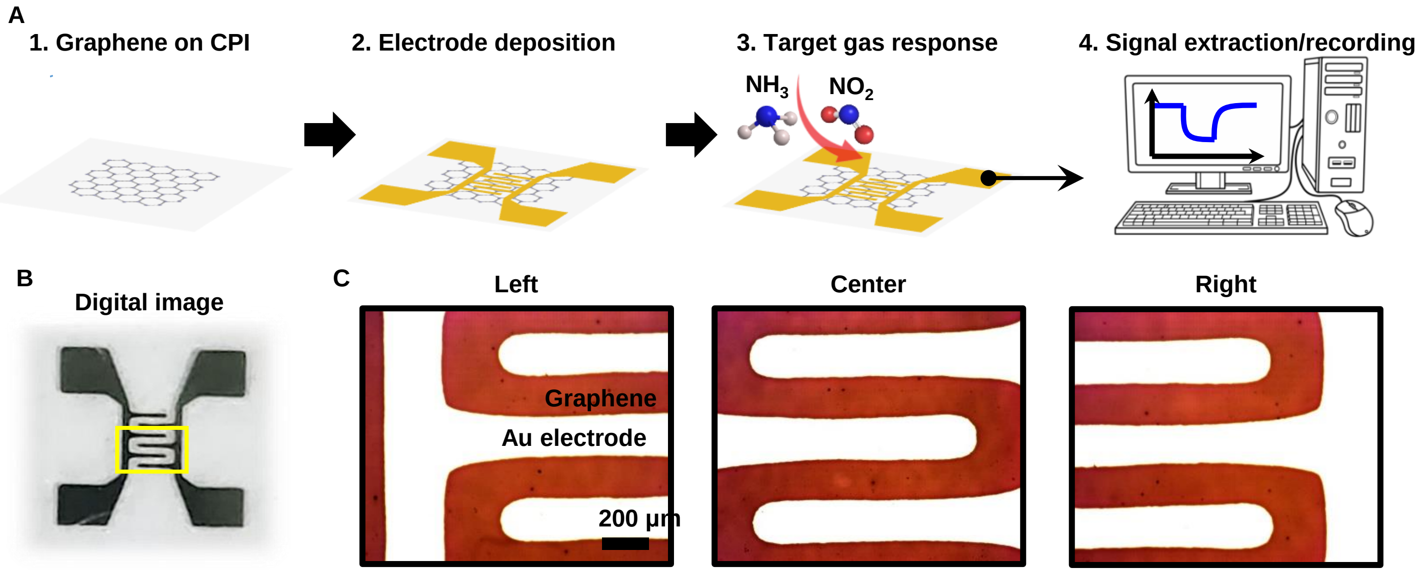

Supplementary Figure 8. (A) Fabrication of the gas sensor for the hybrid microflier application. **(B)** A digital image of the fabricated gas sensor. **(C)** Optical images of each part of the gas sensor in the yellow box of **(B)**.

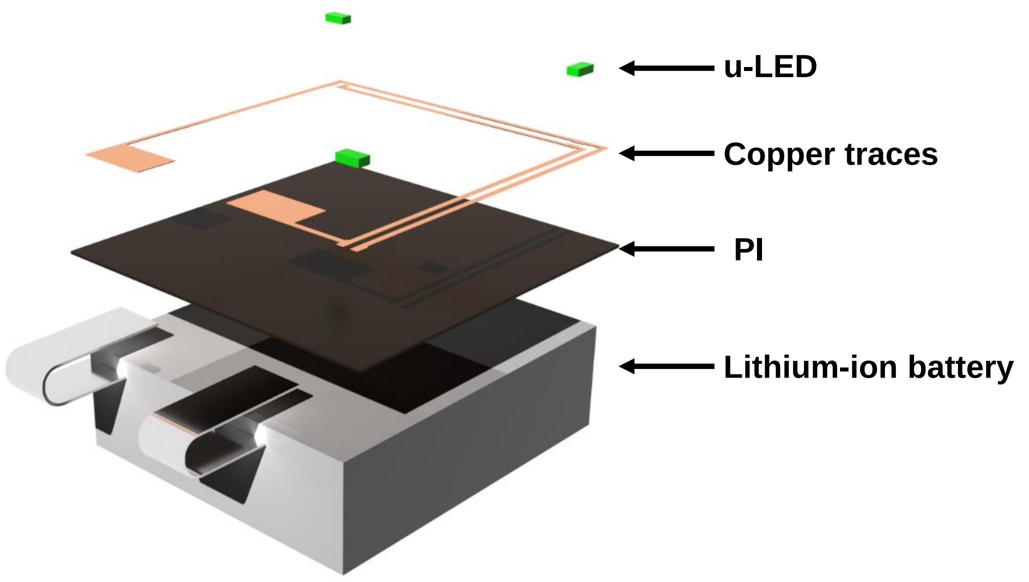

Supplementary Figure 9. Exploded view illustration of the layer and the operation highlights a miniaturized u-LED, connected to copper traces with a battery for wireless power supplies.

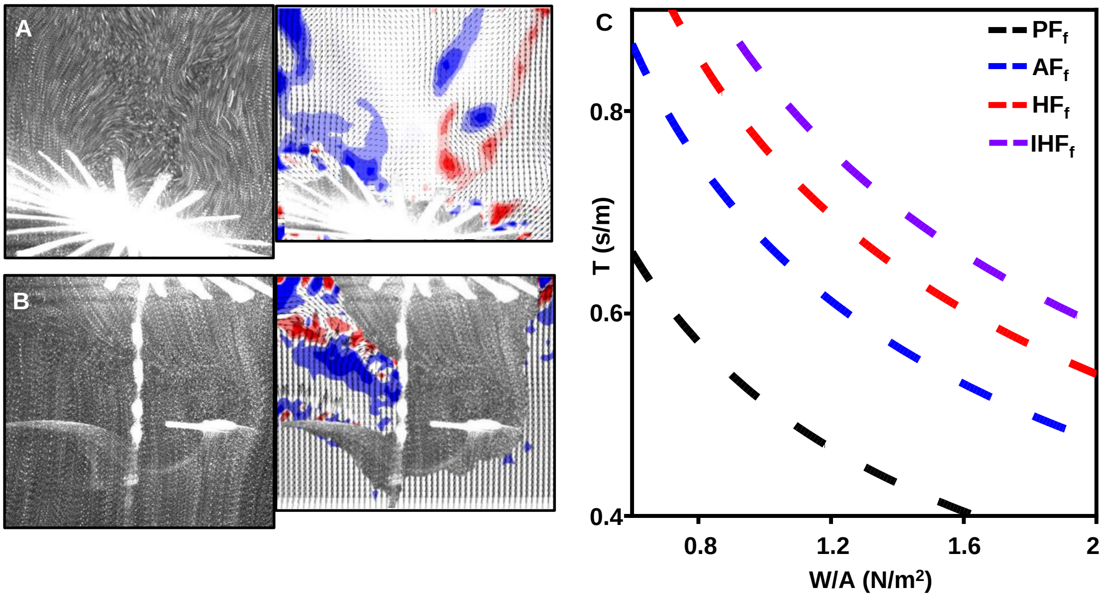

Supplementary Figure 10. Superimposed images of the flow visualization above an improved PF (A, left) and AF (B, left) demonstrating SRV and LEV, respectively as well as corresponding vorticity fields (right). (C) descent time (T) vs wing loading (W/A) of IHF via PTV; corresponding inverse squared relations $T \propto \gamma/\sqrt{W/A}$ (dashed lines); γ is the descent factor.

Captions for videos

Video 1. Free falling of PF, PPF (two parachuting fliers), AF, AAF (two autorotating fliers), Inverse HF (autorotating flier on the top) and HF (from left to right).

Video 2. Flow visualization of (left) PF, (middle) AF, and (right) HF above the wind tunnel.

Video 3. Turbulent wake behind fliers. 3D visualization of vorticity (Q-criteria) in the wake colored by air speed; (left) AF, (middle) PF, and (right) HF via CFD

Video 4. Free-falling experiments of a LED payload (left) with and (middle) without a HF (right) in the dark.
