# Supplementary material for: Functional bio-inspired hybrid fliers with separated ring and leading edge vortices
Source: PNAS Nexus. 2024 Mar 11;3(3):pgae110. doi: 10.1093/pnasnexus/pgae110 (PMC10957237; doi:10.1093/pnasnexus/pgae110)
Supplement: pgae110_Supplementary_Data [file pgae110_supplementary_data.zip › PNASNEXUS-PNASNEXUS-2023-01179R-s02.docx]

# **CFD Simulation Results**

# We perform large eddy simulations (LES) of fliers with fully resolved geometric details to assess their aerodynamic performance. Three cases are studied: (i) a single autorotating flier; (ii) a single parachuting flier; and (iii) a hybrid setup comprising of a parachuting flier at 2D (D is the flier diameter) behind the autorotating flier. The LES model is implemented within a parallel in-house code using the finite element method. Considering the large Reynolds number of the aerodynamics around fliers, we employ residual-based variational multi-scale as a subgrid-scale model, and weak enforcement of essential boundary conditions acts as a near wall. To handle the rotating motion of fliers, we impose the LES model on a boundary-fitted moving mesh via an arbitrary Lagrangian-Eulerian technique. The combination of these advanced numerical methods, which have been validated for a wide range of flow problems, ensures the fidelity and accuracy of the LES simulations.

# Simulation setup

We use cylindrical domains meshed with unstructured tetrahedral elements to perform the LES simulations. The domain size is large enough to eliminate the boundary wall effects. Besides, we design a refinement region in the meshes to better capture the turbulent wake. To simulate the hybrid case, we adopt a multi-domain approach for the autorotating flier and the parachuting flier, wherein the turbulent wake generated from the autorotating flier is projected onto the inlet of the parachuting flier domain. The inlet of the parachuting mesh is placed 1.5D downstream from the autorotating mesh. Consistent projection is utilized to ensure continuous and accurate data transfer between two domains. To present some details of the mesh, we show a cross-section of the meshes of the autorotating flier, parachuting flier, and hybrid setup, respectively, in Extended Data Fig. 4C-E**.** The mesh statistics for all three cases are tabulated in Table 1.

The unidirectional inflow speed of 1.11 m/s is kept constant throughout the three cases. The autorotating flier rotates at $91.1 rad/s$ whereas the parachuting flier remains stationary. The setup for the simulations is depicted in Extended Data Fig. 4A-B. The fluid density and viscosity are based on air at room temperature. We use a time step of $1e-4 s$ for all three cases. The simulations are performed in parallel using Message Passing Interface (MPI) on the Illinois Campus Cluster at the University of Illinois at Urbana-Champaign.

**Table 1 Mesh Statistics for 3 cases**

|  | Single autorotating flier mesh | Single parachuting flier mesh | Hybrid mesh setup |
| --- | --- | --- | --- |
| Minimum element size, m | 1.5e-4 | 1.5e-4 | 1.5e-4 |
| Maximum element size, m | 0.02 | 0.02 | 0.02 |
| Number of nodes | 411,780 | 429,666 | 411,780+(397,804) |
| Number of elements | 2,424,076 | 2,543,368 | 2,424,076+(2,341,566) |
| Note: The additional parachute flier mesh for the hybrid simulation in given in parentheses. | | | |

# CFD Simulation Discussion

We visualize the wake behind the fliers in Extended Data Fig. 5 for the cases of single autorotating flier, single parachuting flier, and hybrid flier setup, respectively at time $t=1.0 s$. The vorticity iso-surface colored by airspeed reveals the complex turbulent structure. The turbulence is further reflected in Extended Data Fig. 6A, which shows the root mean square (RMS) of the velocity fluctuation along the centerline. For all three cases, it is evident that the velocity fluctuations are highest right behind the flier and approach zero further downstream. Generally, we observe that the fluctuations are maximum for the autorotating flier and minimal for the parachute flier. Extended Data Fig. 6B shows the time history of drag coefficients for all three cases. It can be observed that the parachute flier in the hybrid simulation demonstrates a significantly lower drag compared to the drag exerted by the single parachute flier. This is attributed to the fact that much of the kinetic energy has been extracted by the upstream flier. As shown in Extended Data Fig. 6B, the turbulent wake and reduced kinetic energy result in a lower mean drag coefficient and a higher fluctuation. Lastly, based on the prediction, the total drag coefficient of the hybrid simulation, the sum of the drag of the upstream autorotating flier and the downstream parachuting flier, is 0.97.
